# Co-functioning of 2AP precursor amino acids enhances 2-acetyl-1-pyrroline under salt stress in aromatic rice (*Oryza sativa* L.) cultivars

**DOI:** 10.1038/s41598-022-07844-7

**Published:** 2022-03-10

**Authors:** N. Renuka, Vitthal T. Barvkar, Zahid Ansari, Chunfang Zhao, Cailin Wang, Yadong Zhang, Altafhusain B. Nadaf

**Affiliations:** 1 Department of Botany, Savitribai Phule Pune University, Pune, 411007 Maharashtra India; 2 The University Polytechnic, Aligarh Muslim University, Aligarh, 202002 India; 3 Institute of Food Crops, Jiangsu Academy of Agricultural Sciences, Jiangsu Rice Engineering Research Centre, National Centre for Rice Improvement (Nanjing), Nanjing, 210014 China

**Keywords:** Molecular biology, Plant sciences

## Abstract

Aromatic rice (*Oryza sativa*) fetches a premium price due to the pleasant aroma. The major aroma compound 2-acetyl-1-pyrroline (2AP) has been found to be enhanced under stress. This condition can be considered to study the genes, precursors, enzymes, and metabolites involved in elevated levels of 2AP biosynthesis. In the present study, 100 mM salt treatment was given to two aromatic rice cultivars Ambemohar-157 (A-157) and Basmati-370 (B-370) at the vegetative stage (VS_3_). After salt treatment, in the leaves, 2AP contents were elevated by 2.2 and 1.8 fold in A-157 and B-370, respectively. Under these elevated 2AP conditions, the precursor amino acids (glutamate, putrescine, ornithine, and proline), their related genes, enzymes, and metabolites (methylglyoxal and γ-aminobutyric acid (GABA) related to 2AP biosynthesis were analyzed. In addition, agronomic characters were also studied. It was observed that the proline content was enhanced in both the cultivars by 29% (A-157) and 40% (B-370) as compared to control. The Δ^1^-pyrroline-5-carboxylate synthetase (P5CS) enzyme activity was increased in salt-treated plants leaf tissue by 31% (A-157) and 40% (B-370) compared to control. The *P5CS* gene expression was enhanced by A-157 (1.8 fold) and B-370 (2.2 fold) compared to control, putrescine content in A-157 and B-370 decreased by 2.5 and 2.7 fold respectively as compared to control. The ornithine decarboxylase (ODC) activity was enhanced in A-157 (12%) and B-370 (35%) over control. Further, *ODC* gene expression was enhanced in both the cultivars A-157 (1.5 fold) and B-370 (1.3 fold). The diamino oxidase (DAO) enzyme activity was increased by 28% (A-157) and 35% (B-370) respectively over control. The GABA content marginally increased over control in both the cultivars namely, A-157 (1.9%) and B-370 (9.5%). The methylglyoxal levels were enhanced by 1.4 fold in A-157 and 1.6 fold in B-370. Interestingly, the enhancement in 2AP in the vegetative stage also helped to accumulate it in mature grains (twofold in A-157 and 1.5 fold in B-370) without test weight penalty. The study indicated that the ornithine and proline together along with methylglyoxal contribute towards the enhancement of 2AP under salt stress.

## Introduction

In aromatic rice (*Oryza sativa*), the fragrance is the major trait that increases its demand in the international market. The rice aroma comprises of more than 200 volatiles and 2-acetyl-1-pyrroline (2AP) is a foremost component for the pleasant aroma^1,2^. The non-functional betaine aldehyde dehydrogenase 2 (*BADH2*) gene leads to accumulate 2AP in aromatic rice^3^. Nowadays, researchers are focusing more on the biosynthesis of 2AP in aromatic rice^4,5^. Initially, proline was only identified as the precursors, later ornithine, glutamate, putrescine, etc. were also found to act as precursors in 2AP biosynthesis^6–8^. The precursors ornithine and glutamate can produce a common metabolite Δ1-pyrroline-5-carboxylic acid (P5C) through enzymes ornithine aminotransferase (OAT), and Δ1-pyrroline-5-carboxylic acid synthetase (P5CS)^9,10^. Apart from this, ornithine is also metabolized to the polyamine putrescine through ornithine diamine oxidase (DAO) that further gets converted to Δ^1^-pyrroline by ornithine decarboxylase (ODC) to produce 2AP. Another possible pathway to synthesize 2AP is through methylglyoxal non-enzymatic reaction directly with Δ^1^-pyrroline-5-carboxylate (P5C)^9,11^.

The environmental factors play a vital role in defining rice aroma^12–14^. It has been reported that soil salinity has a positive correlation with 2AP synthesis in aromatic rice^15,16^. In the leaves of aromatic rice, salinity elevated 2AP concentration^13^. If the aromatic rice plant is exposed to salt stress during tillering, it increases the 2AP level in the grains^17,18^ however, yield is reduced^19^. Poonlaphdecha et al.^18^ studied the effect of salinity at different growth phases in aromatic rice and revealed that during the vegetative and reproductive phase, it enhances 2AP but significantly reduces the crop yield. Thus, from the previous reports, it’s clear that if the aromatic rice is treated with salt stress during the vegetative phase, it enhances 2AP in the leaves as well as in the grains, but the enzymatic and molecular mechanisms related to 2AP enhancement and the related gene expression have rarely been studied^20^. Therefore, in the present study, 100 mM salt treatment was given to two aromatic rice cultivars Ambemohar-157 (A-157) and Basmati-370 (B-370) at the vegetative stage (VS_3_). After salt treatment, in the leaves, 2AP contents were elevated by 2.2 and 1.8 fold in A-157 and B-370, respectively. Under these elevated 2AP conditions, the precursor amino acids (glutamate, putrescine, ornithine, and proline), their related genes, enzymes, and metabolites (methylglyoxal and γ-aminobutyric acid (GABA)) related to 2AP biosynthesis were analyzed.

## Results

### Optimization of concentration and period of salt treatment for optimal 2AP synthesis in A-157

**Figure 1:**
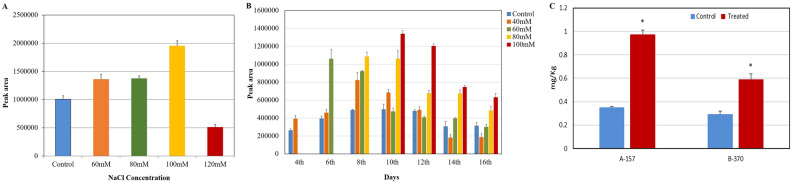
Optimization of (**A**) NaCl concentration, (**B**) period of treatment at 100 mM NaCl for maximum 2AP synthesis in the leaf tissue of A-157 rice cultivar (**C**) Quantification of 2AP in salt-treated plants leaf tissue in aromatic rice cultivars.

Among the treatments (40, 60, 80, 100, and 120 mM NaCl), 100 mM concentration showed optimal 2AP synthesis in terms of its peak area hence this concentration was selected for the determination of the period of salt treatment (Fig. 1A). For this, fresh pots of seedlings were again raised following the above procedure and treated with salt with a gradual increment of 20 mM salt until it reached the desired concentration of 100 mM. For every alternate day, leaf tissue was harvested and 2AP was analyzed in replicates till the 16th day of treatment. Amongst these, the 10th day showed maximum 2AP synthesis in terms of its peak area hence, this time point was selected as an optimum period of treatment (Fig. 1B). In order to create the optimal condition for maximum 2AP synthesis, optimization of salt treatment is necessary. This optimal condition provides an opportunity to study 2AP related genes, precursors, and metabolites.

### Quantification of 2AP in leaves at 100 mM salt treatment

Under the optimized conditions, in A-157 and B-370, 2AP content increased by 2.2 and 1.8 fold respectively over control (Fig. 1C). It has been reported that 2AP content increased significantly under saline conditions during the vegetative stage in aromatic cultivars^16,18^.

### Quantification of precursor amino acids, their enzyme assays, and related gene expression analyses under optimal 2AP synthesis

#### Glutamate and proline

**Figure 2:**
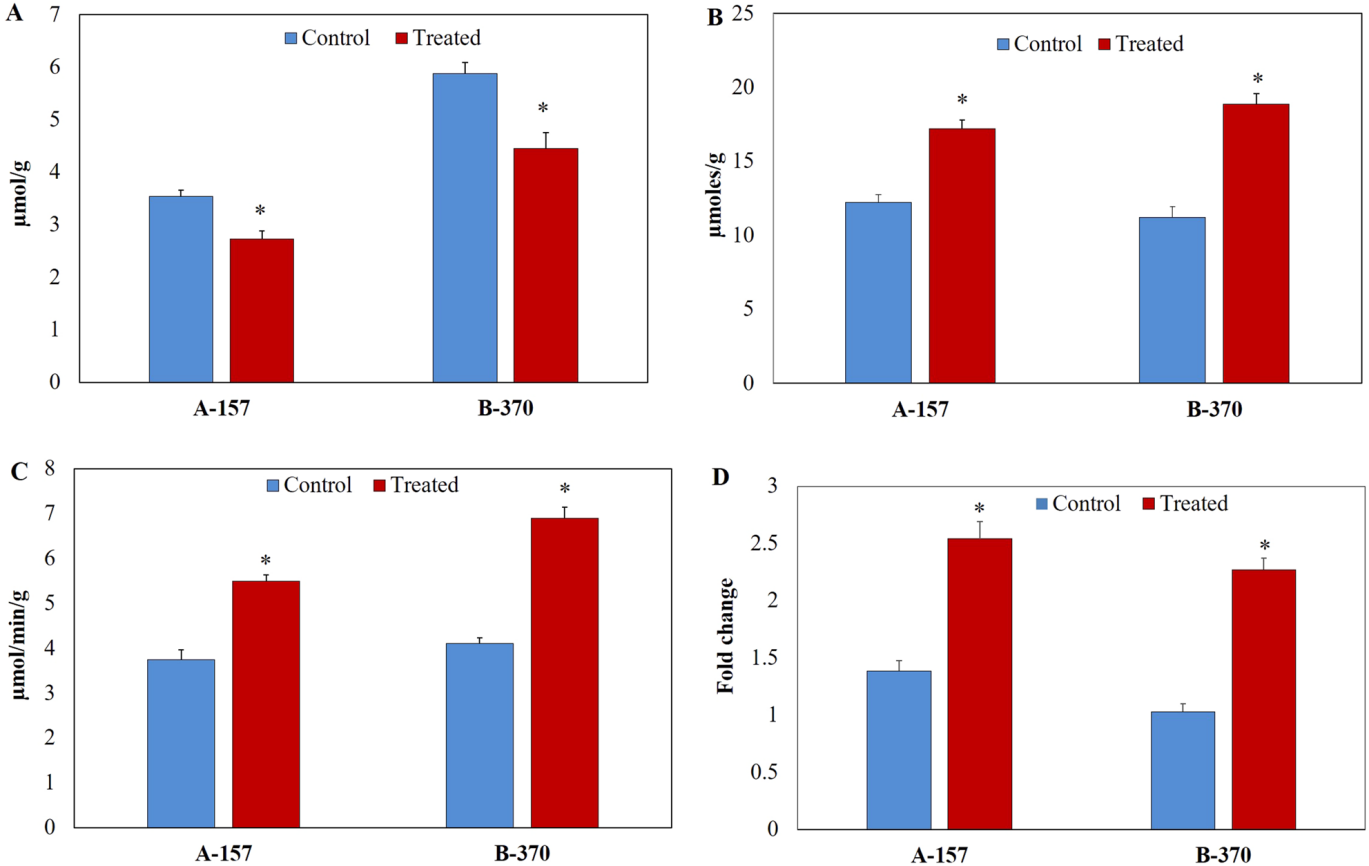
Determination of (**A**) Glutamate, (**B**) Proline content, (**C**) P5CS enzyme assay and (**D**) *P5CS* gene expression in the leaf tissue of aromatic rice cultivars grown under salt stress.

Under the optimized conditions, the glutamate content significantly decreased in both the rice cultivars by 29% (A-157) and 32% (B-370) compared to control (Fig. 2A). However, under the same treatment, proline content was significantly enhanced in leaf tissue in both the cultivars by 29% (A-157) and 40% (B-370) compared to control (Fig. 2B). The increment in proline can be correlated with the P5CS enzyme activity and gene expression. P5CS enzyme activity was increased significantly in both the cultivars by 31% (A-157) and 40% (B-370) compared to control (Fig. 2C). The *P5CS* gene expression was significantly enhanced in A-157 (1.8 fold) and B-370 (2.2 fold) over control (Fig. 2D). A similar reduction in glutamate content was reported in detached leaves under water stress^21^. It has been reported that under salt stress conditions in leaves of aromatic rice proline get accumulated^16,18,22^. The enhanced expression of the *P5CS* gene was reported in salt-stressed aromatic seedlings^23^.

#### Ornithine

**Figure 3:**
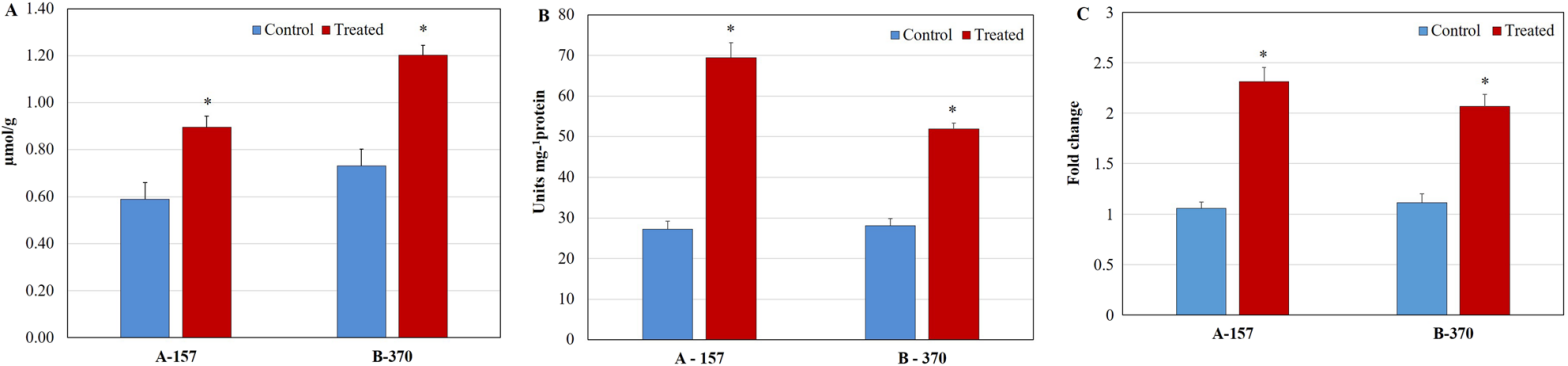
Estimation of (**A**) Ornithine content (**B**) OAT enzyme assay (**C**) *OAT* gene expression in leaf tissue of aromatic rice cultivars grown under salt stress.

Under the optimized conditions, ornithine content was increased significantly in A-157 and B-370 by 19% and 35% respectively in the leaves (Fig. 3A). The OAT enzyme activity was elevated by 60% and 45% in A-157 and B-370 respectively over control (Fig. 3B). The *OAT* gene expression was significantly increased by 2.1 and 1.8 fold in A-157 and B-370 respectively in the leaf tissue (Fig. 3C). An increment in ornithine content under stress was reported in rice under water stress^21^. Other than rice, increased OAT activity in salt-stressed cashew plants and, proline accumulation during salinity was also correlated with increased OAT activity^24^. The increased expression of the *OAT* gene was observed in salt-treated radish cotyledons^25^ and *Arabidopsis thaliana* seedlings exposed to 200 mM NaCl^26^. Banerjee et al.^23^ reported increased expression of the *OAT* gene in aromatic cultivars under salt stress conditions.

#### Putrescine

**Figure 4:**
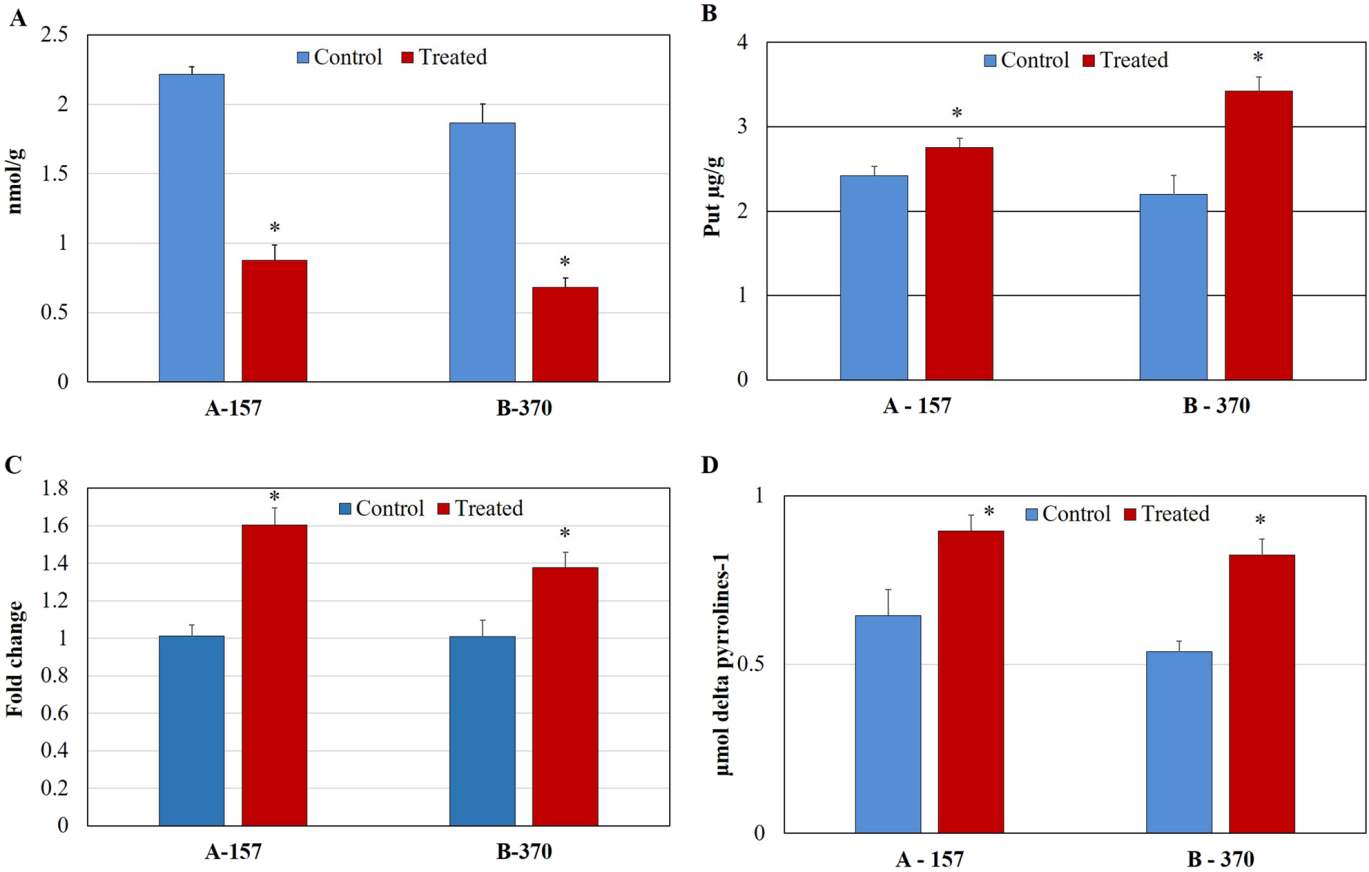
Quantification of (**A**) Putrescine (**B**) ODC enzyme (**C**) *ODC* gene expression and (**D**) DAO enzyme in the leaf tissue of aromatic rice cultivars grown under control and salt stress.

Under the optimized conditions, the putrescine content in both the cultivars A-157 and B-370 significantly decreased by 2.5 and 2.7 fold respectively in the leaves compared to control (Fig. 4A). The ODC activity was significantly enhanced in A-157 (12%) and B-370 (35%) over control (Fig. 4B). Further, *ODC* gene expression was enhanced in both the cultivars A-157 (1.5 fold) and B-370 (1.3 fold) (Fig. 4C). Banerjee et al.^16^ also observed a drastic reduction in putrescine content in aromatic rice seedlings under salt stress.

#### DAO

Under the optimized conditions, the DAO enzyme activity was significantly increased by 28% (A-157) and 35% (B-370) respectively over control (Fig. 4D). Banerjee et al.^23^ reported enhancement in the *ODC* gene expression in two varieties under salt stress conditions.

#### BADH2

**Figure 5:**
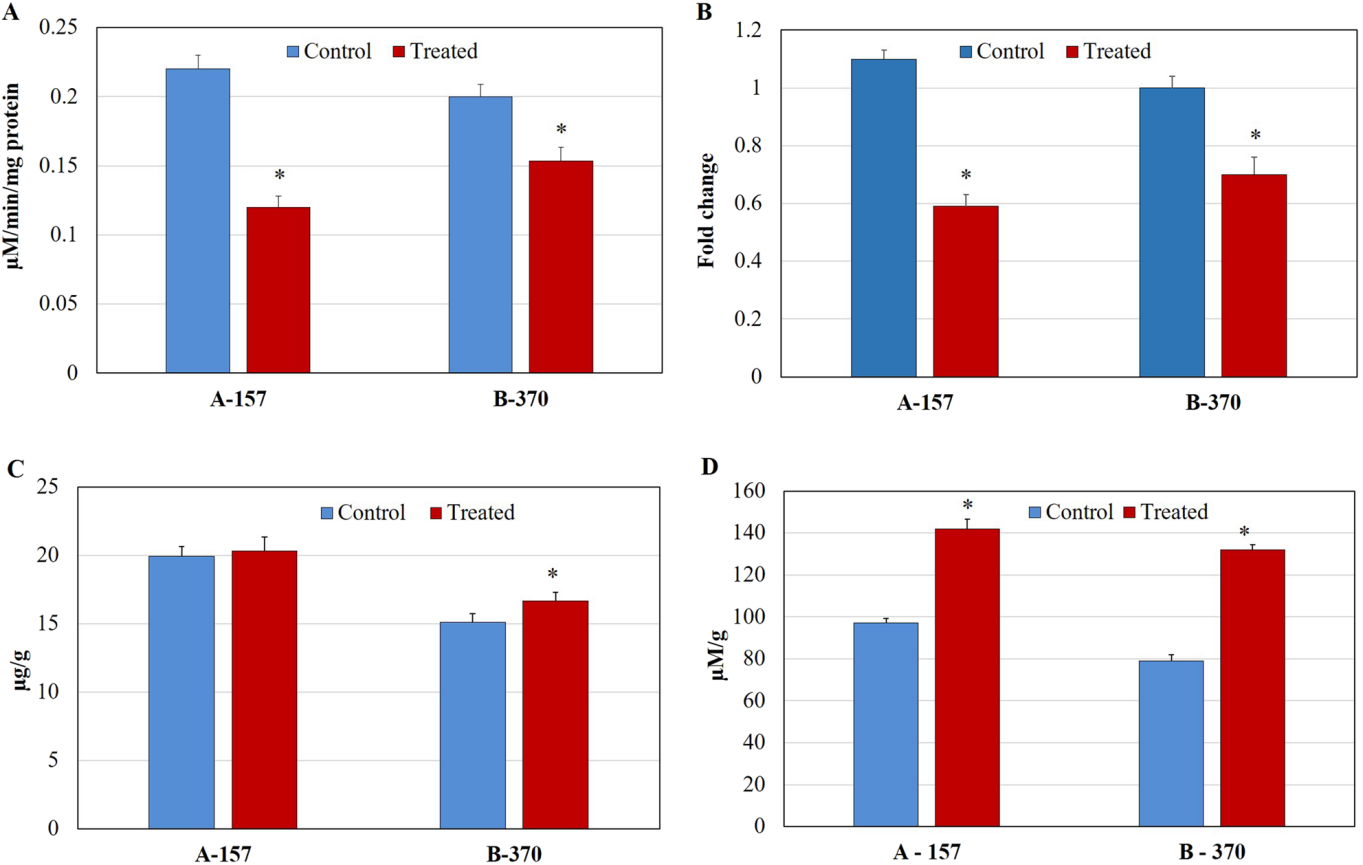
Analysis of (**A**) BADH2 enzyme assay (**B**) *BADH2* gene expression (**C**) GABA (**D**) Methylglyoxal in leaf tissue of aromatic rice cultivars grown under control and salt stress.

Under the optimized conditions, BADH2 enzyme activity was significantly decreased in the leaf tissues in both the cultivars by 45% and 23% in A-157 and B-370 respectively over control (Fig. 5A). The *BADH2* gene expression was decreased significantly by 1.8 and 1.4 fold in A-157 and B-370, respectively (Fig. 5B). It is in agreement with the previous report of Banerjee et al.^16^. In their recent study, they observed reduced expression of the *BADH2* gene under salt stress in four aromatic varieties from West Bengal^23^.

#### GABA

Under the optimized conditions, GABA content differentially increased in both the cultivars over control. In A-157 (1.9%) the increment was non-significant whereas, in B-370 (9.5%) it was significant (Fig. 5C). GABA content was reported to enhance significantly under salt stress during initial vegetative and reproductive phases^18^. Further, in the latest report, increased GABA content in the seedlings of non-aromatic and also some aromatic rice varieties were observed^16^.

#### Methylglyoxal

Under the optimized conditions, the methylglyoxal levels were significantly enhanced in the leaf tissue by 1.4 fold in A-157 and 1.6 fold in B-370 (Fig. 5D) over control. Similar observations were reported under saline conditions in aromatic rice varieties^16^.

#### 2AP in mature grains

**Figure 6:**
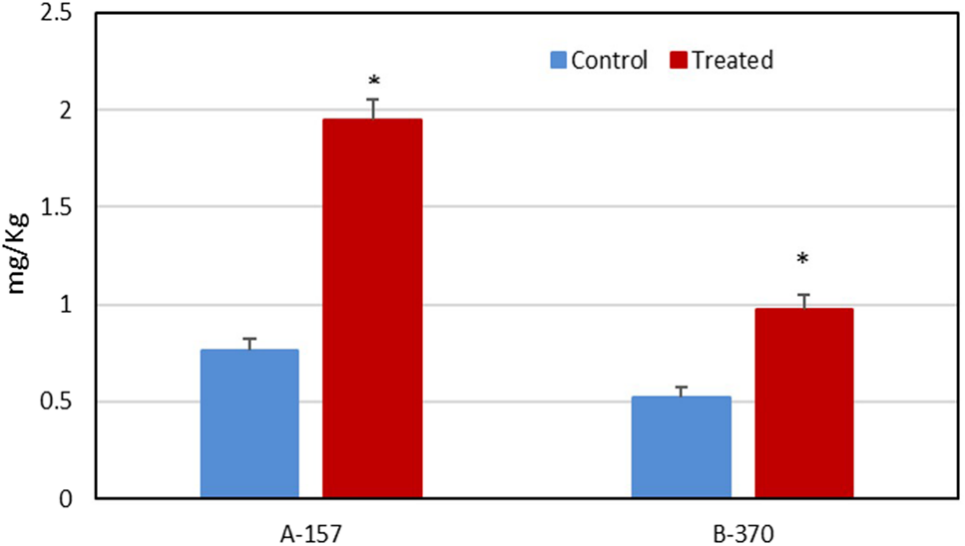
Quantification of 2AP in mature grains of aromatic rice cultivars under salt stress.

Under the optimized conditions, the 2AP content in seeds was significantly increased by twofold in A-157 and 1.5 fold in B-370 over control (Fig. 6). Gay et al.^17^ also observed that when aromatic rice cultivars were grown in the field with salt stress increases 2AP in grains. Poonlaphdecha et al.^18^ reported significant increases in 2AP content in the grains when aromatic rice plants were treated with NaCl.

### Assessment of phenotypic traits in the salt-treated cultivars

In both the cultivars, under the optimized conditions, panicle length and spikelet percentage significantly reduced after treatment, whereas, in B-370, plant height, test weight, and the number of grains were significantly reduced. The number of productive tillers, length, and breadth of the grains didn’t change significantly in salt-treated seedlings in both the cultivars (Table 1). It is reported that when salt stress was induced during the vegetative phase, test weight was not significantly reduced^18^. In Aychade aromatic rice, salt stress induced during the vegetative phase did not induce a severe effect in agronomic traits^18^.

**Table 1:** Assessment of phenotypic traits in the cultivars under salt treatment.

Agronomic characters	A-157		B-370	
	Control	Treated	Control	Treated
Plant height (cm)	115 ± 5.0	107 ± 6.1	104 ± 3.6	89 ± 7.1**
Panicle length (cm)	24.06 ± 0.8	21.26 ± 1.13*	24.43 ± 2.5	19.8 ± 1.2*
Number of tillers	5.66 ± 0.4	5.33 ± 0.3	5.33 ± 0.3	5.33 ± 0.4
Number of grains	164.67 ± 15.3	141.33 ± 12.5	60.33 ± 3.0	50.18 ± 4.3*
Number of filled grains	136.45 ± 12.4	108.21 ± 8	48.18 ± 4.1	36.28 ± 2.1
Spikelet percentage	82.86 ± 9	76.57 ± 9.1*	79.86 ± 2.9	72.30 ± 7.5*
Test weight (g)	12.67 ± 0.8	12.03 ± 0.3	11.35 ± 0.3	10.34 ± 0.4**
Length of grain (mm)	4.61 ± 0.07	4.59 ± 0.09	6.88 ± 0.09	6.87 ± 0.04
Grain breadth (mm)	2.02 ± 0.04	2.02 ± 0.03	1.97 ± 0.05	1.96 ± 0.02

*Significant at *p* = 0.05 level.

**Significant at p = 0.01.

### Correlation analysis

**Figure 7:**
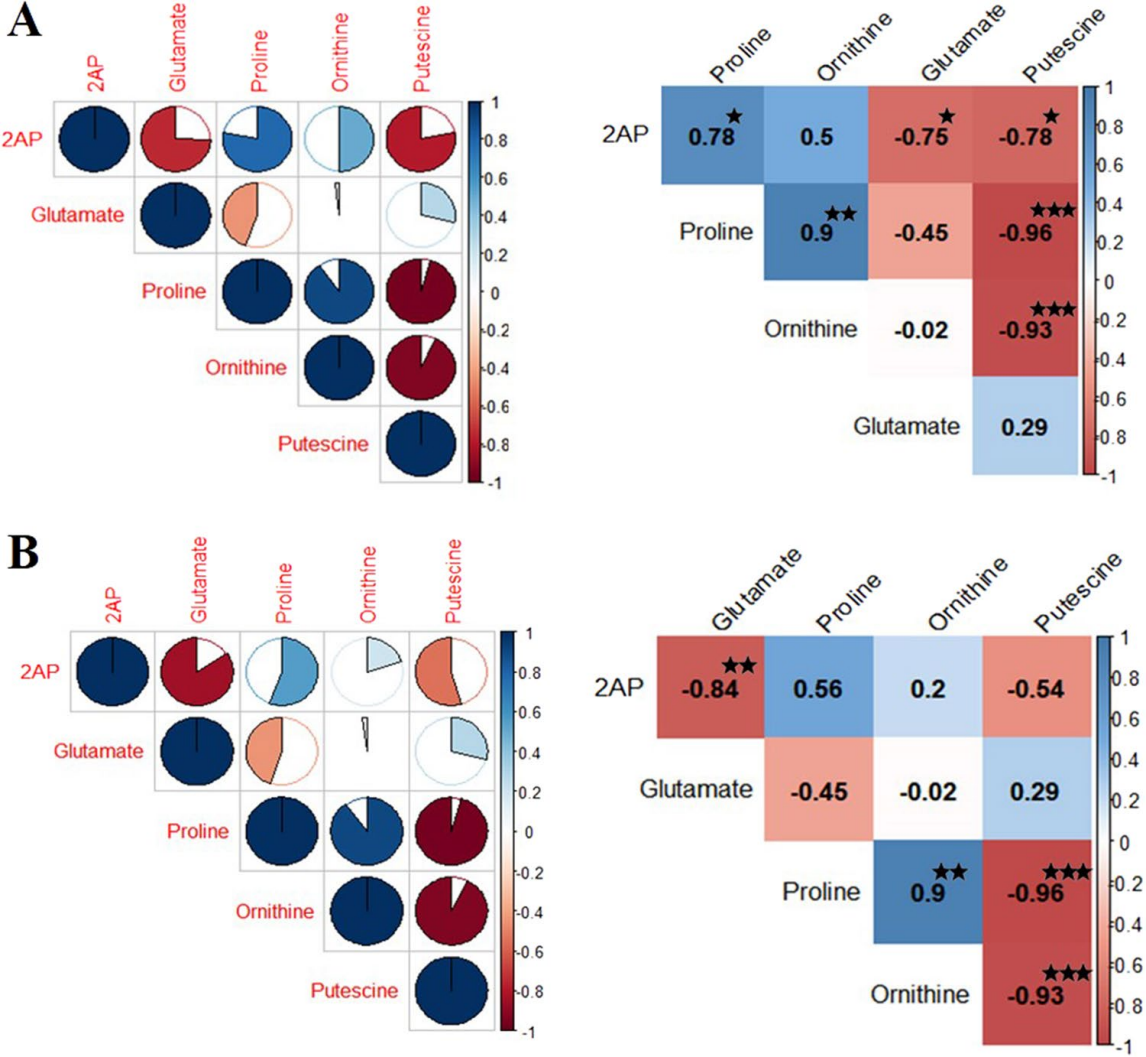
(**A**) Correlation analysis of precursor amino acids with 2AP at vegetative stage (**B**) Correlation analysis of precursor amino acids with 2AP in mature grains.

The correlation analysis between precursor amino acids and 2AP at vegetative as well as mature stages of growth indicated a positive correlation of 2AP with proline and ornithine and a negative correlation with other amino acids (Fig. 7A,B). This indicates the involvement of ornithine and proline towards 2AP synthesis at both stages. The correlation analysis also supports the upregulation of these amino acids and their related enzymes and genes observed in the present study.

## Discussion

The glutamate is an osmoregulator that occupies a central position in the plant’s primary metabolism^27^. The carbon skeleton and α-amino group of glutamate form the basis for the synthesis of proline, GABA, and arginine. In plants, proline synthesis takes place through the glutamate pathway and ornithine pathway. Under osmotic stress, proline is accumulated through the glutamate pathway. The proline is synthesized from glutamic acid via intermediate ∆^1^-pyrroline-5-carboxylate (P5C). The reaction is being catalyzed by ∆^1^-pyrroline-5-carboxylate synthetase (P5CS) and ∆^1^-pyrroline-5-carboxylate reductase (P5CR)^28^. It is reported that higher expression of the *P5CS* gene led to a significant increase in proline content in transgenic aromatic rice varieties^29^. In the present study, low levels of glutamate are recorded under salt stress, on the other hand, P5CS enzyme activity and expression levels increased with the significant increase in proline. This suggests that the pool of glutamate in both varieties might have diverted towards the synthesis of proline leading to decreased glutamate content. As far as, *P5CS* gene expression is concerned, it is reported to be higher in aromatic rice cultivars at all the developmental stages compared to non-aromatic rice^30^.

Proline is an osmoprotectant and its accumulation is a common physiological reaction under biotic and abiotic stresses in plants^31^. In plants, proline metabolism has mainly been studied in response to abiotic stress and its accumulation plays adaptive roles in plant stress tolerance. The most prevalent pathway of proline synthesis is through glutamate; alternative pathways of proline synthesis are through ornithine^4,32^. A higher level of proline content was reported in aromatic rice cultivars under all the developmental stages compared to non-aromatic rice^30^. Enhancement of proline content in grains is associated with increased 2AP content in grains^33,34^. Under salt stress, the enhanced pool of proline is further utilized for 2AP synthesis as seen in the present study.

Ornithine, a non-protein amino acid, plays an important role in sensory, essential in the regulation of plant growth and development; helps in regulating molecule which is involved in producing proline and polyamines^35,36^. In rice callus, the activity of the OAT enzyme was found to be enhanced compared to non-aromatic rice callus^9^. Yang et al.^21^ reported that higher content endogenous ornithine is associated with water stress encouraged proline accumulation in detached rice leaves. In this study ornithine content and associated enzyme activity of OAT and gene expression was increased under salt stress. Ornithine can be transaminated to P5C by OAT that leads to the synthesis of proline^20^. In *A*. *thaliana*, ornithine accumulation increased the enzyme and gene expression of *OAT* which leads to the accumulation of proline^26^. In our studies, in addition to proline, ornithine levels were also increased, showing its positive correlation with proline under salt stress. Ornithine is metabolized into putrescine via the ODC enzyme which is further converted to Δ^1^-pyrroline^37^. Under salt stress conditions, putrescine can be used to synthesize Δ^1^-pyrroline upon catalysis by DAO along with the generation of ammonia and hydrogen peroxide^38,39^. We have found that under salt stress, the enzyme and gene expression activities of both these enzymes are enhanced along with increased Δ^1^-pyrroline. Thus, it can be said that the increased levels of ornithine might be responsible for elevating the activities of these enzymes. However, putrescine levels did not increase which might quickly get converted to Δ^1^-pyrroline through DAO. 2AP production in grains of aromatic rice is associated with DAO and soluble protein^5,10^. Thus, our study highlights the enhanced expression of ornithine along with proline under salt stress.

The elevation of acetyl donor Δ^1^-pyrroline results in increased 2AP synthesis in aromatic rice^40^. Supplementation of Δ^1^-pyrroline to rice callus enhanced 2AP content in both aromatic and non-aromatic cultivars^41^. Δ^1^-pyrroline formed via proline, glutamate, and ornithine ultimately undergoes non-enzymatic reactions with methylglyoxal to form 2AP^42^.

Methylglyoxal is a cytotoxic by-product generated from glycolysis^42^. In aromatic rice, methylglyoxal interacts non-enzymatically with Δ^1^-pyrroline to synthesize 2AP^38^. Under abiotic stress, methylglyoxal content is increased drastically^43,44^. In this study increased methylglyoxal content was observed under salt stress conditions that might be contributing to enhanced 2AP levels in both cultivars.

The non-functional *BADH2* gene which encodes an amino aldehyde dehydrogenase is responsible for the elevation of 2AP in aromatic rice. In non-aromatic rice cultivars of *japonica*, Chinese and *indica* rice; the *BADH2* gene was down-regulated by RNAi which enhanced 2AP synthesis in transgenic mature leaves and grains^45–47^. The BADH2 transcript was much higher in the non-aromatic IR-64 as compared to that in the aromatic cultivars under either control or stress conditions. Under salt stress conditions in aromatic rice seedlings, *BADH2* gene expression was found to be decreased^23^. Our results are in agreement with these reports.

In aromatic rice, 2AP is the major compound for fragrance^48^. It has also been reported that stress during cultivation, such as drought and salinity stress led to higher 2AP content in rice grains^7,17,18^. Mild drought stress at tillering and booting stage in aromatic rice enhanced 2AP content in brown rice^34^. In A-157 and B-370, 2AP was increased under salt stress conditions in leaf tissue and mature grains. Recently, it has been reported that even seed priming and soaking in saline condition enhanced 2AP content in aromatic rice varieties^49^. In our studies also 2AP has been enhanced under salt stress that supports these reports.

GABA, a non-protein amino acid, involved in the various biochemical process and also a signaling molecule in plants^48,50^. It is synthesized via *BADH2* in rice. However, in aromatic rice, since the *BADH2* gene is non-functional, a reduction in its levels was expected. But salt stress conditions might be triggering other pathways that are contributing towards GABA. Synthesis of GABA through glutamate via GAD enzyme is one of the ways.

Yield improvement is one of the aims to overcome the increasing demand of aromatic rice. Aromatic rice cultivars produce significantly less yield compared to non-aromatic cultivars^51,52^. A significant impact on the yield of cereal crops was reported under stress. Certainly, the 2AP biosynthetic pathway is genetically controlled, nevertheless, agronomic practices can also affect the agronomic traits, enzyme assays, and genes involved in 2AP biosynthesis^53^. It has been reported that yield is reduced in rice crops when grown under saline conditions^19^. Later, Poonlaphdecha et al.^18^ reported that when salinity is induced during the reproductive phase it affects test grain weight and during the vegetative phase it has not affected the test grain weight in aromatic rice. In our study, salt stress was induced during the vegetative stage so test grain weight was not affected in both cultivars. Thus, salt treatment during the vegetative stage might have less impact on test weight with the added advantage of aroma in grains.

**Figure 8:**
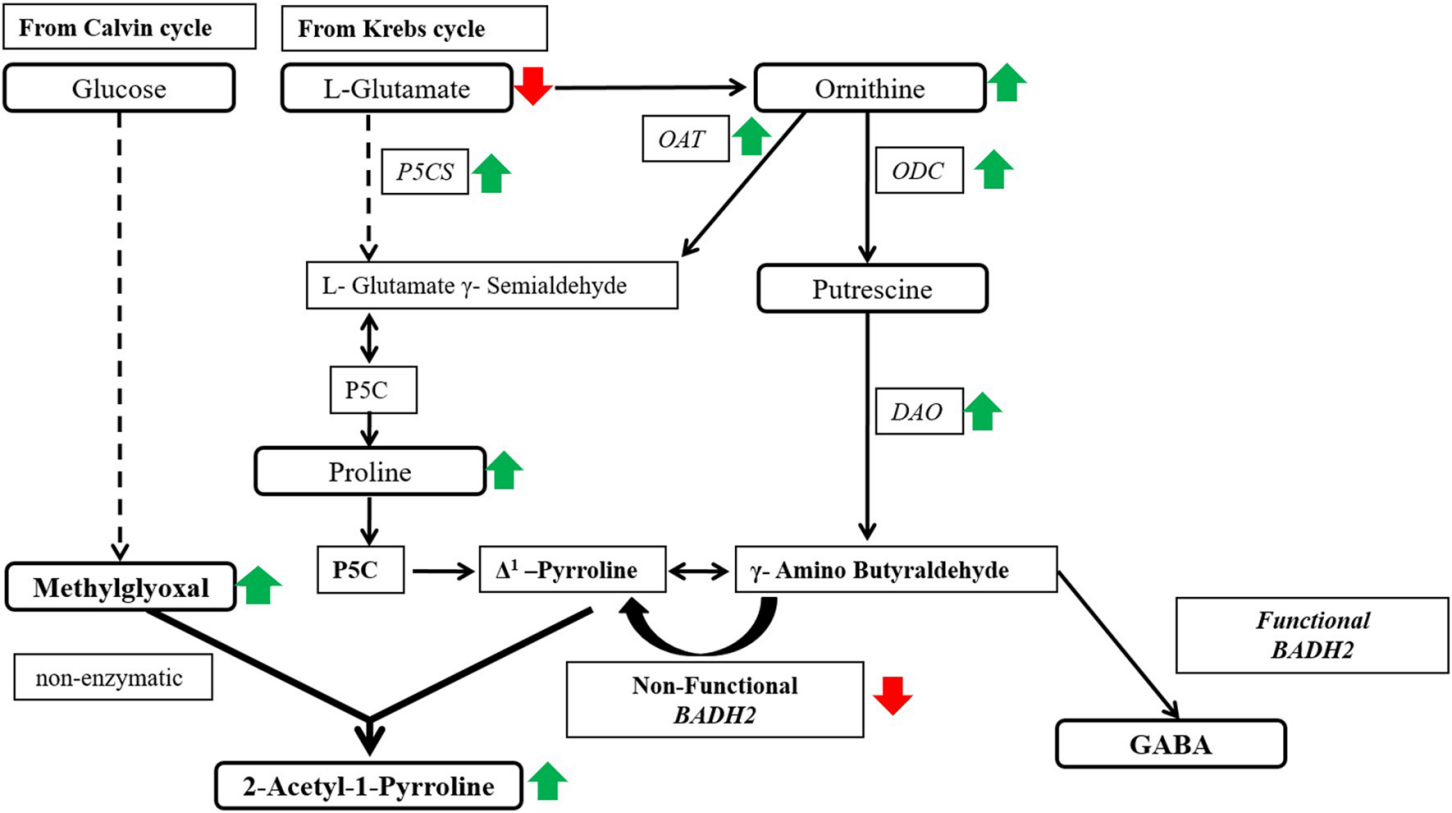
2AP biosynthetic pathway in aromatic rice under salt stress. *P5CS* Δ^1^-Pyrroline-5-carboxylic acid synthetase, *OAT* Ornithine aminotransferase, *ODC* Ornithine decarboxylase, *DAO* Diamine oxidas, *P5C* Δ^1^-Pyrroline-5-carboxylic acid, *BADH2* Betaine aldehyde dehydrogenase, *GABA*: γ-Aminobutyric acid. Red up arrow indicates that it has enhanced and reduced indicated by green down arrow.

Recently biosynthesis of 2AP in aromatic rice has been the main focus of research^4,5^. Some amino acids and metabolites like glutamate, proline, ornithine, putrescine, and methylglyoxal which get provoked under stress conditions are involved in the synthesis of 2AP through Δ^1^-pyrroline^38^. Under salt stress, enhanced 2AP conditions were created that provided an opportunity to analyze the possible precursor amino acids, related genes, enzymes, and metabolites to understand the 2AP biosynthetic pathway in a better way. The study indicated that the precursor amino acids ornithine and proline go hand in hand and contribute towards the enhancement of 2AP which are supported by methylglyoxal; the methyl ketone donor of 2AP (Fig. 7A,B). The enhancement in 2AP takes place in the vegetative stage that helps to accumulate it in mature grains without test weight penalty. Thus, short-duration salt treatment at the vegetative stage helps in the enhancement of 2AP in grains. The biosynthetic pathway under salt stress depicts that the amino acids proline and ornithine are the major contributors along with methylglyoxal in the synthesis of 2AP under salt stress. The other amino acids glutamate and putrescine might contribute towards the synthesis of proline. The upregulation of these amino acids through their respective genes helps to synthesize 2AP at the vegetative stage and finally to deposit it in the grains (Fig. 8).

## Conclusions

The short-duration salt treatment at the vegetative stage enhances 2AP and helps in better understanding of 2AP pathway. Under salt stress, more than one precursor amino acids contribute towards 2AP enhancement in grains without severe effect on phenotypic traits. This study will help further to understand the co-functioning of 2AP related genes.

## Materials and methods

### Procurement of aromatic rice cultivars and raising the seedlings

For the present study, two cultivars A-157 and B-370 were selected. A-157 is a non-basmati type aromatic short grain rice cultivar popularly cultivated in the Pune district of Maharashtra state of India. B-370 is the basmati type long-grain cultivar popularly cultivated in the Jammu region of North India. The seeds of A-157 and B-370 were obtained from the Rice Research Station, Vadgaon Maval, Pune, Maharashtra, India. The seedlings were raised in a polyhouse at the Botanical garden, Savitribai Phule Pune University, Pune, Maharashtra, India. Seeds of both the cultivars were soaked in tap water for 24 h followed by incubation at room temperature in a wet muslin cloth for 48 h. Pre-germinated rice seeds were sown in trays filled with soil and allowed to grow for 21 days. After 3 weeks, seedlings were transplanted in pots (45 × 21 × 15 cm) containing soil and farmyard manure (3:1). The pots were arranged in a randomized block design.

### Optimization concentration of salt and period of treatment

After 20 days of transplantation, the seedlings of A-157 were treated with various concentrations of salt (40, 60, 80, 100, and 120 mM) with an increment of 20 mM every alternate day until the desired concentration was reached. The control seedling pots were watered with fresh water. The control and salt-treated pots were arranged in triplicates and watered with an equal quantity of water. When the seedlings showed wilting symptoms, the leaf tissue was harvested and 2AP was analyzed in replicates. Among the treatments, 100 mM concentration showed optimal 2AP synthesis hence this concentration was selected for the determination of the period of salt treatment. For this, fresh pots of seedlings were again raised following the above procedure and treated with salt with a gradual increment of 20 mM salt until it reached the desired concentration of 100 mM. For every alternate day, leaf tissue was harvested and 2AP was analyzed in replicates till the 16th day of treatment. Amongst these, the 10th day showed a maximum 2AP synthesis hence, this time point was selected as an optimum period of treatment.

The same period and concentration of salt were applied to another cultivar B-370. The leaf tissue was harvested in liquid Nitrogen at this time point and stored at − 80 °C for further analyses of metabolites, enzyme activity, and gene expression analysis along with control leaves. 8 pots per cultivar from this experimental set were continued further for studying agronomic characters and 2AP in mature grains. For this, the salt treatment was discontinued, and pots were watered with fresh water until harvesting.

### Quantification of 2AP in salt-treated plants leaf tissue and grains

Headspace-solid phase micro-extraction (HS-SPME) (Supelco, Bellefonte, PA, USA) coupled with gas chromatography-flame ionized detector (GC-FID) (Shimadzu 17A, Japan) was used for quantification of 2AP in salt-treated plants leaf tissue and grains. For this, extraction and quantification of 2AP from leaves and grains were done following the optimized protocol from our laboratory^30^.

### Quantitative estimation of metabolites in salt-treated plants leaf tissue

#### Glutamate, ornithine, proline, putrescine, methylglyoxal, and GABA

In A-157 and B-370 control and treated leaves, all the analysis was done. Estimation of glutamate, sample preparation was done following the method of Valle et al.^54^ and quantification was done using high-pressure liquid chromatography (HPLC) (Agilent Technologies, 1260 Infinity, UA) following the protocol of Hill et al.^55^. Ornithine content was estimated following the method of Chinard^56^. Proline content was estimated following the method of Bates^57^. Methylglyoxal level was determined following the method of Yadav et al.^58^. GABA was quantified using the method described by Karladee and Suriyon^59^. Putrescine was quantified following the method of Redmond and Tseng^60^.

### Enzyme activity

The P5CS enzyme activity was done by following the standard method of Kavi kishor et al.^61^. BADH2 enzyme activity in leaves of A-157 and B-370 was analyzed by Bradbury et al.^3^. OAT activity was assayed according to Chen et al.^40^. Assay to determine the enzyme activity of ODC was done following the spectrophotometric method of Legaz et al.^62^. DAO activity was measured according to the method of Holmstedt et al.^63^.

### RNA extraction and cDNA synthesis

Total RNA was isolated from control and treated leaf samples of A-157 and B-370 using Sigma RNA isolation kit. Total RNA was treated with DNase and quantified using nanodrop (NanoDrop™ 1000 Spectrophotometer). The first strand of cDNA was synthesized using iScriptTM cDNA synthesis kit (Bio-Rad, USA) using 1 μg of total RNA.

### Quantitative real-time PCR (qRT-PCR)

For qRT-PCR, primers for the genes under study and housekeeping gene, *elongation factor 1-alpha* (*EF1α*) were designed using the DNA sequences from NCBI database (Table 2.) qRT-PCR was performed in a real-time PCR machine (Real Plex, Eppendorf, Germany) using SYBR Green Brilliant® II qPCR master mix (Stratagene, USA). The qRT-PCR reaction was performed in a total volume of 10 μl containing 1X Fast SYBR® Green qPCR master mix (Biorad, USA), 1 μl of primer (10 pmol/μl) and 1 μl cDNA. Thermal cycling conditions were kept as follows: initial denaturation at 95 °C for 30 s, 57 °C for 30 s, and 72 °C for 30 s. *EF1α* was used as control and relative expressions of genes were measured following the 2^−ΔΔCT^ method^64^. Primer sequence details are given in (Table 2).

**Table 2:** Details of the genes and their primers used for gene expression analysis.

Sr. No	Gene	Primer sequence (5′–3′)
1	*P5CS* F	GAAGTGGTAATG GTCTTCTC
	*P5CS* R	AGCAAATCTGCGATCTCATC
2	*BADH*2 F	TGTGCTAAACATAGTGACTGGA
	*BADH*2 R	CTTAACCATAGGAGCAGCT
3	*ODC* F	ACGAGGTGGTGAGGGGTTAT
	*ODC* R	ACCCGTTGAAGTCTGAGCTG
4	*OAT* F	GACCGATTTCATCCAATTCAAGA
	*OAT* R	ACACCTCCTTGTTGCGATGT
5	*EF1*α F	TTTCACTCTTGGTGTGAAGCAGAT
	*EF1*α R	GACTTCCTTCACGATTTCATCGTAA

### Agronomic characterization in salt-treated aromatic rice cultivars

The seedling height and number of productive tillers per plant were recorded. The panicles were harvested and the length of the panicle, the number of filled/unfilled grains per panicle, grain length and breadth, and test weight were recorded.

### Statistical analysis

All the experimental data values are of three replicates, and the results are presented as mean ± standard error. The significance test was done by Student’s t-test at *p* ≤ 0.05. The data visualization and analysis were done using the R software tool (version 4.0.0) on R-studio integrated development environment (version 1.3.959). For data visualization ‘ggplot2’ library was used, and correlation analysis was done using the libraries ‘corrplot’ and ‘corrgram’.

### Statements

This is to state that the collection of seed material and field experiments carried out in the present study was in accordance with relevant guidelines framed by the IUCN Policy Statement on Research Involving Species at Risk of Extinction and the Convention on the Trade in Endangered Species of Wild Fauna and Flora. The necessary permission was obtained by the authors for the collection of seed material from the Rice Research Station of Vadgaon Maval, Pune.
